# Canadian Pharmacists Association: Transforming Primary Care in Canada Summit 2024: White paper

**DOI:** 10.1177/17151635241285920

**Published:** 2024-10-12

**Authors:** Shania Liu, Danielle Paes, Yazid N. Al Hamarneh, Ross T. Tsuyuki

**Affiliations:** EPICORE, Department of Medicine, Faculty of Medicine and Dentistry, University of Alberta, Edmonton, Alberta; Canadian Pharmacists Association, Ottawa, Ontario; EPICORE, Department of Medicine, Faculty of Medicine and Dentistry, University of Alberta, Edmonton, Alberta; Department of Pharmacology, Faculty of Medicine and Dentistry, University of Alberta, Edmonton, Alberta; EPICORE, Department of Medicine, Faculty of Medicine and Dentistry, University of Alberta, Edmonton, Alberta; Department of Medicine, Faculty of Medicine and Dentistry, University of Alberta, Edmonton, Alberta

## Background and introduction

Primary care is a model of care that serves as the foundation of the health system, underpinning Canadians’ access to care for everyday and lifelong health care needs. Primary care encompasses care for common ailments, mental and psychosocial health, maternity and childcare, home care, and disease prevention, assessment and management.^1^ The World Health Organization (WHO) highlighted the following as the core functions of primary care^2^:

- **First contact accessibility** creates a strategic entry point for and improves access to health services.
- **Continuity** promotes the development of long-term personal relationships between a person and a health professional or a team of providers.
- **Comprehensiveness** ensures that a diverse range of promotive, protective, preventive, curative, rehabilitative and palliative services are provided.
- **Coordination** organizes services and care across levels of the health system and over time.
- **People-centred** care ensures that people have the education and support needed to make decisions and participate in their own care.

Similar concepts have also been articulated as they relate to primary care and pharmacy practice in a recent editorial.^3,4^

On June 6, 2024, primary care key stakeholders, including family physicians, nurses, pharmacists, policy-makers, pharmacy technicians, student representatives and patient advocates, were brought together by the Canadian Pharmacists Association (CPhA) to discuss the transformation of primary care in Canada. This forum aimed to achieve a common vision: a vision of a patient-centred, collaborative and integrated system that positions pharmacy as a first point of contact for primary care—a vision that leverages the education, skills and expertise of all health practitioners to deliver high-quality and accessible care to everyone in Canada. The summit concluded with the release of a communiqué^5^ that highlighted recommendations and key areas that need to be addressed to better support and leverage pharmacists in primary care.

*“Pharmacists are primary care providers: a first point of contact for primary care.”*

*[Danielle Paes, chief pharmacist officer, Canadian Pharmacists Association]*

## The perfect storm

*Moderator and panellists: Glen Doucet (CEO, Canadian Pharmacists Association), Dr. Jean-Joseph Condé (board member, Canadian Medical Association), Kimberley Hanson (CEO, Health Partners), Dr. Treena Klassen (chair, Specialty Nursing Council, Canadian Nurses Association), Dr. Danielle Paes (chief pharmacist officer, Canadian Pharmacists Association).*

The scene was set during the opening roundtable discussion, in which the current state and pressing challenges in primary care in Canada were explored by leaders representing medicine, nursing and pharmacy, as well as persons with lived experience. The nation’s primary care landscape was described as “the perfect storm” in which growing demands on the system due to an aging population, increasing comorbidities and complexity in required care are not being adequately met by a decreasing supply of system resources, in part influenced by a diminishing workforce of family physicians and nurse practitioners who are retiring, leaving the profession or experiencing burnout.^6,7^ This has led to more than 6.5 million Canadians, or 1 in 5, living without access to a regular family physician.^8^ The squandering of existing resources was identified as an additional factor contributing to pressure on the health system. Pharmacists’ roles, responsibilities and scope in primary care need to be better defined, maximized and communicated broadly to all health practitioners, patients and the government. Likewise, pharmacy technicians and assistants should be encouraged and enabled to practise to their full abilities and education. The use and standardization of access to health technology and health information, such as health record access by patients and health practitioners, were also identified key areas requiring attention. The result of these factors is a primary care system that is in a state of crisis: an overloaded health system in which Canadians cannot receive the primary care they need in a timely manner. As illustrated by Kimberley Hanson, CEO of Health Partners, “Emergency rooms have increasingly become walk-in clinics,” as a result of such challenges in accessing primary care. However, crisis can also lead to opportunity. During the summit, several areas were identified as potential opportunities for pharmacists and pharmacy teams to be better positioned and integrated as a first point of nonurgent care, not only to improve the efficiency of the health system but, ultimately, to increase access to the care that so many patients currently need.

*“Emergency rooms have increasingly become walk-in clinics.”*

*[Kimberley Hanson, CEO, Health Partners]*

### Democratizing health data and technology

During the same panel, challenges in accessing health data, by patients and health practitioners, and differences in the accessibility of that data among health practitioners were recognized as key barriers to efficient health care. It was proposed that concerns around patients’ abilities to interpret their own health data should be replaced with increased efforts to encourage patient education and empowerment. Efforts to arm patients with their own health information will allow them to better advocate for themselves and enable them to engage with their health care providers to make more informed decisions about their health. Standardization of access to health data across health practitioners would support interprofessional collaboration, enable all providers to effectively use their knowledge and skills and allow for delivery of a higher level of primary care across the board. The development of novel technology to support patient care through seamless and secure information sharing and integration of systems within everyday practice should be a priority. Furthermore, the removal of regulatory barriers restricting the use of health technology and automation may facilitate increased system efficiency and redirection of pharmacists’ time toward the provision of care.

## Benchmarking scope of practice

*Moderators and panellists: Allison Bodnar (CEO, Pharmacy Association of Nova Scotia), Pam Kennedy (pharmacist and owner, Bridgewater Guardian Pharmacy), Sheila MacLeod (senior executive director, Benefits Programs, Province of Nova Scotia Department of Health and Wellness), Beverley Zwicker (registrar and CEO, Nova Scotia College of Pharmacists).*

Pharmacists’ scope of practice represents a key opportunity to transform primary care in Canada. Pharmacies are one of the main first points of entry into the health care system. As such, enabling pharmacists to practise to the full scope of their education and training improves patients’ access to a broader spectrum of primary care services, including preventative health screening, chronic disease management, medication management, administration of vaccines and injectable medicines, the management of common ailments and the provision of health education.

To demonstrate what this may look like in practice, an evaluation of the new Community Pharmacy Primary Care Clinics in Nova Scotia was presented as a case study. Primary care services being delivered by pharmacists practising at their full scope across Nova Scotia have proven to be highly successful and, in many instances, have prevented the need for hospital emergency room visits. Furthermore, pharmacists’ opportunity to practise to their full scope was shown to improve professional satisfaction, confidence, knowledge and skills. Limitations include the additional time, financial, administrative and human resources required to deliver these services. To help overcome these challenges, government support was seen as a strong enabler of pharmacists practising to their full scope. Benchmarking of pharmacists’ scope of practice across all jurisdictions in Canada is an imperative step to drive the transformation of the primary care landscape.

*“The evolution of pharmacy practice and primary care is important because it is a critical part of redesigning the health care system to meet the needs of Canadians today and into the future—a system that provides better care, not Band-Aid care.”*

*[Beverley Zwicker, registrar and CEO, Nova Scotia College of Pharmacists]*

## Strengthening chronic disease management

*Moderators and panellists: Dr. Ross Tsuyuki (professor of medicine, Faculty of Medicine and Dentistry, University of Alberta), Dr. Yazid Al Hamarneh (assistant professor, associate director and scientific officer, University of Alberta), Dr. Christine Papoushek (pharmacotherapy specialist, University Health Network—Toronto Western Family Health Team), Anne Marie Picone (interim executive director, New Brunswick Pharmacists’ Association).*

Primary care is a pillar for chronic disease prevention and management. However, the growing burden of chronic disease is placing unprecedented pressure on the nation’s health care system. Chronic diseases affect more than 44% of Canadians over the age of 20,^9^ with a projected increase in rates of 14% annually.^10^ Inadequate management of chronic diseases continues to place a significant burden on the health care system, leading to more than 89% of all deaths^11^ and $80 billion in annual health costs in Canada.^12^ As such, urgent attention is needed to address the growing demands on primary care and the nation’s health care system.

Pharmacists’ interventions in chronic disease are well-supported by high-level evidence in the literature. For instance, a recent meta-analysis of studies examining the effectiveness of hypertension interventions performed by different health care teams showed that pharmacist interventions led to the largest reduction in blood pressure (adjusted, −7.3 mmHg [95% CI, −9.1 to −5.6]), compared with nurse-led (−3.0 mmHg [95% CI, −4.2 to −1.9]) and physician-led (−2.4 mmHg [95% CI, −3.4 to −1.5]) interventions, which had significant but much lower effects.^13^ Despite this evidence, there remains the need for research on the implementation of these interventions into practice and to better educate the public about the role pharmacists play in the prevention and management of chronic diseases.^14^ Key ways to achieve this include building relationships and rapport with our patients and other providers, practising interprofessional collaboration, focusing care on priority areas for the local population, integrating pharmacy teams into provincial and jurisdictional health planning and strategies and supporting pharmacists to practise to their full scope. For example, a strategy to facilitate pharmacists’ delivery of care to their full scope has been developed in Alberta, where pharmacists can obtain additional prescribing authority. An innovative Primary Care Pathway summarizing the evidence and clinical guidelines into an electronic algorithm is a proposed mechanism to support pharmacists in the clinical assessment, management, follow-up and documentation of prevalent chronic diseases such as cardiovascular disease.

Terminology is vital when discussing our profession among ourselves or describing it to others. There are certain terms we should not use, such as “minor ailments”, “allied health professionals”, “clients” or “customers”.^15^

*“Terminology matters—there are certain terms we will not use, such as ‘minor ailments’, ‘clients’ or ‘customers’.”* ^15^

*[Yazid Al Hamarneh, assistant professor, associate director and scientific officer, University of Alberta]*

## Building the pharmacy workforce of the future

*Moderator and panellists: Dr. Lisa Dolovich (professor and dean, Leslie Dan Faculty of Pharmacy, University of Toronto), Ayman Lakhani (PharmD candidate and VP Education, Canadian Association of Pharmacy Students and Interns), Michelle Owen (executive director, HHR Task Force, Strategic Policy Branch, Health Canada), Clara Wong (professor, Pharmacy Technician Program, Centennial College).*

Like other health professions, there is a growing shortage of pharmacists. Innovative strategies are urgently needed to address the pharmacist workforce at both the influx and efflux points. For instance, recruitment and training of more pharmacists and pharmacy technicians are required. These efforts should be accompanied by the removal of barriers to enter the profession, such as high levels of tuition and licensing pathways for International Pharmacy Graduates. Efforts to improve pharmacists’ work satisfaction (such as enabling pharmacists to practise autonomously to the full extent of their education as discussed previously) may reduce burnout and attrition. Pharmacy technicians and assistants working to the full scope of their training may alleviate pharmacists’ workload and also reduce burnout. The “Pharmacist First” model, in which the pharmacist first conducts a clinical evaluation and education before a technician fills the prescription, is an example of a workflow model in which technicians can support pharmacists in delivering efficient, patient-centred care.^16^ Finally, pharmacists, similar to their interprofessional colleagues, should be encouraged and better supported (e.g., financially incentivized) to work in rural and remote areas of Canada, where residents often face the greatest challenges in accessing health care.

*“As we redesign primary care, the systems we design need to not only be patient-centric, but they need to be patient-led.”*

*[Kimberley Hanson, CEO, Health Partners]*

## Are all stakeholders at the table?

*Moderator and panellists: Joelle Walker (vice president, Public and Professional Affairs, Canadian Pharmacists Association), Avis Favaro (senior advisor, Santis Health), Stephanie Gawur (principal, Santis Health), Nikita James Nanos (chief data scientist, Nanos Research Corporation).*

As we pave the path forward, non-tokenistic representation of all stakeholders at tables informing health policy is key. Advocacy bodies representing key stakeholders should work as a unified front in an interprofessional and patient-driven manner. This may be achieved through collaboration between key health practitioner associations (such as the Canadian Medical Association, Canadian Nurses Association, Canadian Pharmacists Association), and, most important, meaningful engagement of patient advocacy groups, to cocreate and achieve clarity on the solutions we need to transform primary care.

*“Every person on medication does not have a doctor, but every person taking medication has a pharmacist.”*

*[Anne Marie Picone, interim executive director, New Brunswick Pharmacists’ Association]*

## Summary

Canada’s primary care crisis is a complex problem requiring a multifaceted solution. Pharmacists are well-positioned to be part of this solution as highly accessible and trusted health providers. As Anne Marie Picone of the New Brunswick Pharmacists’ Association summarized, “Every person on medication does not have a doctor, but every person taking medication has a pharmacist.” During this Primary Care Summit, several opportunities for action to help transform our health care system to ease the current primary care crisis were highlighted. These included recognition of pharmacy as a first point of primary care, innovative models of care involving the entire pharmacy team practising to the full scope of their knowledge and training, supporting the pharmacy workforce across education levels and geographic locations, better integration of health data and pharmacy teams in primary care to improve the efficiency of the health system, and an interprofessional, patient-driven approach to advocacy. Evidence to inform how pharmacies can contribute towards transforming primary care is clear.^13,17,18^ As a profession, it is no longer about getting our foot in the door—the door is wide open. Pharmacists and pharmacy teams need to champion themselves as a key facet in the redesign of the health care system to better meet the needs of Canadians today and into the future.

*“As a profession, it is no longer about getting our foot in the door—the door is wide open.”*

*[Glen Doucet, CEO, Canadian Pharmacists Association]*
